# Fine Mapping for Identification of Citrus Alternaria Brown Spot Candidate Resistance Genes and Development of New SNP Markers for Marker-Assisted Selection

**DOI:** 10.3389/fpls.2016.01948

**Published:** 2016-12-23

**Authors:** Jose Cuenca, Pablo Aleza, Andres Garcia-Lor, Patrick Ollitrault, Luis Navarro

**Affiliations:** ^1^Centro de Citricultura y Producción Vegetal, Instituto Valenciano de Investigaciones AgrariasValencia, Spain; ^2^CIRAD, UMR AGAPPetit-Bourg, France

**Keywords:** mandarin, fungal disease resistance, gene mapping, molecular markers, *Alternaria alternata*

## Abstract

Alternaria brown spot (ABS) is a serious disease affecting susceptible citrus genotypes, which is a strong concern regarding citrus breeding programs. Resistance is conferred by a recessive locus (*ABSr*) previously located by our group within a 3.3 Mb genome region near the centromere in chromosome III. This work addresses fine-linkage mapping of this region for identifying candidate resistance genes and develops new molecular markers for ABS-resistance effective marker-assisted selection (MAS). Markers closely linked to *ABSr* locus were used for fine mapping using a 268-segregating diploid progeny derived from a heterozygous susceptible × resistant cross. Fine mapping limited the genomic region containing the *ABSr* resistance gene to 366 kb, flanked by markers at 0.4 and 0.7 cM. This region contains nine genes related to pathogen resistance. Among them, eight are resistance (R) gene homologs, with two of them harboring a serine/threonine protein kinase domain. These two genes along with a gene encoding a S-adenosyl-L-methionine-dependent-methyltransferase protein, should be considered as strong candidates for ABS-resistance. Moreover, the closest SNP was genotyped in 40 citrus varieties, revealing very high association with the resistant/susceptible phenotype. This new marker is currently used in our citrus breeding program for ABS-resistant parent and cultivar selection, at diploid, triploid and tetraploid level.

## Introduction

Alternaria brown spot (ABS) is a fungal disease caused by the tangerine pathotype of *Alternaria alternata* (Fr.) Keissl., that induces necrotic lesions on fruits and young leaves, defoliation and fruit drop in susceptible citrus genotypes (Akimitsu et al., 2003). The disease was first observed in Australia in 1903 on “Emperor” mandarin (Pegg, 1966) and was subsequently detected in citrus-growing regions all over the world (Vicent et al., 2000; Timmer et al., 2003; Golmohammadi et al., 2006; Wang et al., 2010). Currently, ABS control is primarily based on fungicide application. Depending on the climate of the region and the susceptibility of the cultivar, between four and ten fungicide sprays per year are needed to produce quality fruit for the fresh market. Even with this large number of sprays, damage reduction is not always satisfactory. These constraints force growers to remove susceptible cultivars such as “Fortune” or “Nova” mandarins (Cuenca et al., 2013). Thus, genetic resistance remains as the best option for disease control (Bhatia et al., 2003; Peres and Timmer, 2006; Vicent et al., 2007).

The tangerine pathotype of *A. alternata* is a necrotrophic pathogen, and carries a gene cluster (*ACTT*) located in a small chromosome responsible for ACT-toxin biosynthesis (Ajiro et al., 2010). This host-specific toxin is released during the germination of conidia, rapidly affecting the plasma membrane integrity of susceptible host cells (Kohmoto et al., 1993). There is also indirect evidence suggesting the presence of toxin receptors in susceptible citrus genotypes (Tsuge et al., 2012).

Due to constraints of the citrus reproductive system, such as polyembryony in most mandarins, specific characteristics or capacity to produce triploid hybrids (Aleza et al., 2010b), several ABS-susceptible cultivars are being used as parents in many mandarin breeding programs, both at diploid and triploid level (McCollum, 2008; Aleza et al., 2010a,b; Cuenca et al., 2010; Grosser et al., 2010; Aleza et al., 2012). This is the case of the monoembryonic cultivar “Fortune,” widely used as female parent in the triploid breeding programs in Spain (Navarro et al., 2012), France and Italy (Recupero et al., 2005; Froelicher et al., 2012); the susceptible cultivars “Murcott” and “Ponkan” are widely used as male parents in many diploid breeding programs carried out in Japan and Brazil (JinPing et al., 2009; Schinor et al., 2012). Also, this is the case for “Dancy,” “Minneola,” “Nova,” “Fairchild,” “Fremont,” “Page,” “Orlando,” “Pixie,” and “Daisy,” which are also used as male parents or for induced mutations in diploid and triploid breeding programs in Spain (Navarro et al., 2012) and USA (Williams, 2012). On the other hand, previous studies stated that the inheritance of ABS resistance in citrus is controlled by a single recessive allele (Dalkilic et al., 2005; Gulsen et al., 2010; Cuenca et al., 2013). Therefore, segregation is expected in progeny arising from crosses between resistant and heterozygous ABS-susceptible parents or even between two heterozygous ABS-susceptible ones.

Genetic mapping of agronomical traits is of principal importance in breeding programs, and its usefulness has been widely demonstrated for marker-assisted selection (MAS) and for candidate gene identification and cloning (Dirlewanger et al., 2004; MacKay and Powell, 2007; Sorkheh et al., 2008). In addition, defining the exact chromosomal position for genes of interest, i.e., fine-mapping, is critical for improving the effectiveness of MAS, since the smaller the distance from the gene controlling the trait, the more accurate will be the selection (Lörz and Wenzel, 2008).

Genetic mapping can be achieved mainly by linkage mapping and/or association mapping. Linkage mapping, in which a progeny segregating for the trait of interest is analyzed, has been a useful approach to map many agronomical traits in several crops (Würschum, 2012), including the mapping of resistance genes to fungal pathogens. In contrast, association mapping studies a collection of genotypes with a broader genetic variation in a more representative genetic background than in linkage-mapping (Ingvarsson and Street, 2011; Khan and Korban, 2012).

In a recent study (Cuenca et al., 2013), we located a region containing the so-called *ABSr* locus, near the centromere on chromosome III using bulked-segregant and half-tetrad analyses from triploid populations. The identified region was flanked by a Simple Sequence Repeat (SSR) marker (TTC8) and a Single Nucleotide Polymorphism (SNP) marker (CiC3248-06), found at 3.77 and 1.71 cM from the *ABSr* locus, respectively, delimiting a 3.3 Mb genome region. Moreover, no recombination was observed between another SSR marker (AT21) and the *ABSr* locus. This locus appears to be included in a genomic region very rich in disease resistance homologous genes.

In the present study, we have developed new SNP markers to perform fine-linkage mapping of the previously located region. The *ABSr* locus has now been restricted to 366 kb, containing several candidate genes that may be involved in ABS-resistance. Moreover, a new SNP marker with very little recombination frequency with the phenotype has been tested for a large set of genotypes used as breeding mandarin parents, demonstrating its usefulness in MAS for a wide range of breeding populations.

## Materials and methods

### Linkage mapping population

A 268-diploid mapping population obtained from a cross between “Fortune” mandarin (*C. clementina* × *C. tangerina*; ABS heterozygous susceptible—“*Aa”*) and a hybrid between clementine and sweet orange –“C × SO-1” (*C. clementina* × *C. sinensis*; ABS resistant—“*aa”*) was used to perform linkage mapping. The cross was made in spring 2011 and seedlings were maintained in the greenhouse during the experiments.

### Phenotyping method for resistance to *Alternaria alternata*

The evaluation of ABS response was assessed by *in vitro* inoculation of young detached leaves from the mapping population, their parental genotypes and controls, following the procedure described by Vicent et al. (2004).

#### Inoculum production

A virulent single-spore isolate of *A. alternata* (IVIA-A005) isolated from an infected “Fortune” mandarin fruit was used for inoculations. Abundant conidia were obtained by a method adapted from Everts and Lacy (1996). The isolate was grown on potato dextrose agar (PDA) plates at 25°C in darkness for 8–10 days and then, illuminated with fluorescent lamps (Philips TLD 18 W/33) at 25°C for 8 h to initiate conidiophore formation, and placed in the dark at 18°C for 12 h. Conidial suspensions were prepared by pouring sterile water over the colonies and gently rubbing the surface with a sterile glass rod. The suspension was filtered through two layers of cheesecloth, and the spore concentration was adjusted to 10^5^ conidia·ml^−1^ with a haemocytometer. Suspensions with conidial germination lower than 90% were discarded.

#### Leaf inoculations

**Figure 1:**
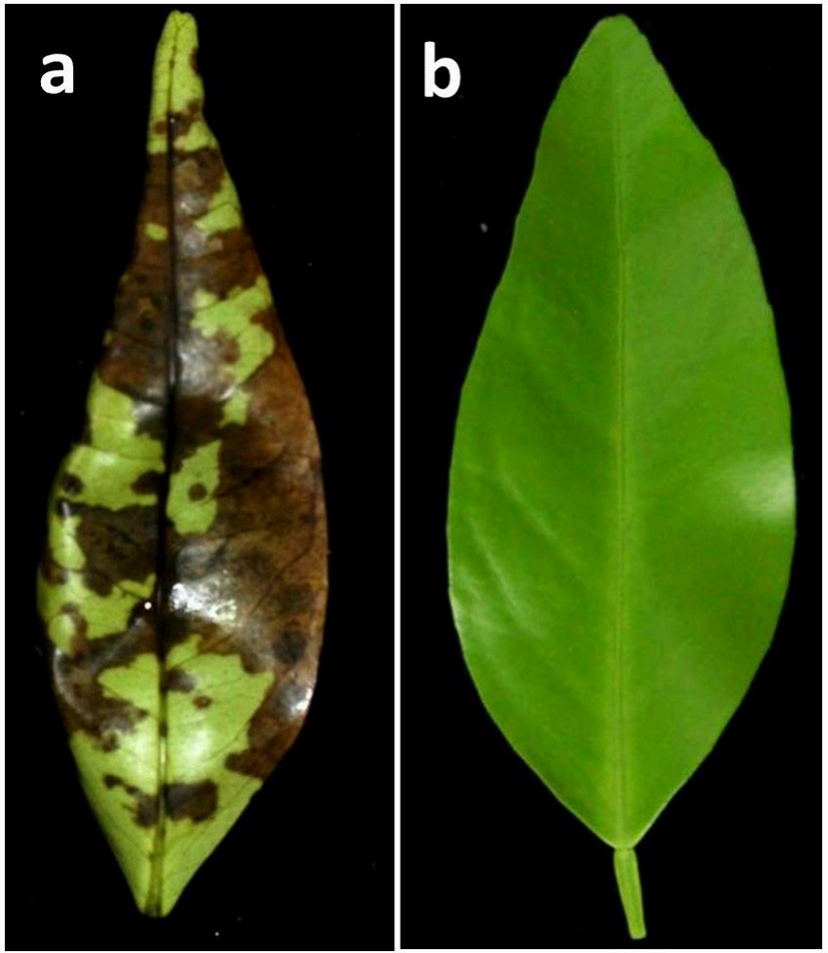
**Symptoms observed 48 h after ***in vitro*** inoculation of young detached leaves from control genotypes. (A)** Susceptible cultivar “Fortune.” **(B)** Resistant cultivar “CXS0-1.”

Bioassays were performed immediately after leaf harvest. Young leaves (about 50% developed) were inoculated with 10^5^ conidia·ml^−1^. This suspension was sprayed over both upper and lower surfaces of each leaflet, using five leaves per genotype. Controls were inoculated by spraying sterile distilled water. Leaves were incubated in a moist chamber in the dark at 27°C. In susceptible genotypes, leaf symptoms appear during the second day after inoculations and very clear necrosis induced by the ACT-toxin can be observed after 48–72 h, when the results were evaluated (Figure 1). A genotype was considered resistant when no symptoms of ABS were observed in any leaf, whereas presence of infection was recorded when a clear symptom of ABS was observed in at least one leaf. The inoculations were repeated when there were doubts regarding interpretation. Experiments inoculating the whole population were carried out twice each spring in 2013, 2014, and 2015.

### DNA isolation

Genomic DNA of the mapping population and germplasm accessions was isolated using the Plant DNeasy kit from Qiagen, Inc. (Valencia, CA, USA), following the manufacturer's protocol. DNA concentrations were estimated with PicoGreen® and adjusted to 30 ng/μl for further analyses.

### Simple sequence repeat (SSR) genotyping

Polymerase chain reactions (PCRs) were performed with wellRED oligonucleotides (Sigma-Aldrich®, St Louis, MO, USA) using the following protocol: Mastercycler ep Gradient S (Eppendorf Scientific Inc., Westbury, NY, USA); reaction volume, 15 μl; 0.8 U Taq polymerase (Fermentas®, Burlington, VT, USA); reaction buffer [750 mMTris-HCl (pH 9), 50 mMKCl, 200 mM (NH_4_)_2_SO_4_, 0.001% bovine serum albumin], 0.1 mM of each dNTP, 5 mM MgCl_2_, 3 mM of each primer and 30 ng of total DNA. The PCR program was as follows: 94°C for 5 min; 40 cycles of 30 s at 94°C, 1 min at 55°C, and 30 s at 72°C; final elongation 10 min at 72°C. Separation was carried out by capillary gel electrophoresis (CEQ 8000 Genetic Analysis System; Beckman Coulter Inc., Fullerton, CA, USA). Data collection and analysis were carried out using the GenomeLabGeXP10.0 software (Beckman Coulter Inc.). The molecular markers used for SSR genotyping were AT21 and TTC8 (Cuenca et al., 2013).

### Fragment sequencing and SNP mining

#### Fragment sequencing

Twenty DNA 600 bp-fragments spanning the *ABSr* region were sequenced in “Fortune” and “C × SO-1” genotypes to find polymorphisms that could be heterozygous (*Aa*) in “Fortune” and homozygous (*aa*) in “C × SO-1.” Information on location of the corresponding sequences on the haploid clementine reference genome (www.phytozome.org/clementine) and annotation is given in Table 1. In order to obtain more information correlated with the expected polymorphisms in relation with the response to the disease, “Clemenules” clementine (*C. clementina*) and “Minneola” tangelo (*C. paradisi* × *C. tangerina*), which are homozygous resistant and homozygous susceptible, respectively (from available segregation data) were also included for fragment sequencing. Twenty-two diallelic SNPs were found useful (heterozygous in “Fortune,” homozygous in “C × SO-1” and “Clemenules” and the alternative homozygous type in “Minneola”). Nine SNPs, included in 9 sequenced fragments, were selected for subsequent analysis.

**Table 1:** **Annotations on the haploid clementine's reference genome (http://www.phytozome.net/clementine) corresponding to the sequenced fragments**.

**#Seq**	**Locus name**	**Position initial**	**Position final**	**Annotation**	**Exon/Intron**
1	Ciclev10023819	24585082	24585682	Aluminum response	Exon
2	Ciclev10018715	24629196	24629796	Glycosylphosphatidylinositol anchor synthesis protein	3′UTR
3	Ciclev10019689	24798391	24798991	Glycine/serine hydroxymethyltransferase	Exon
4	Ciclev10021676	24838352	24838952	Amino acid tRNAsynthetase	Exon
5	Ciclev10019906	24898091	24898691	Cysteine protease required for autophagy—Apg4p/Aut2p	5′UTR
6	Ciclev10022372	24943349	24943949	large subunit ribosomal protein L9e	3′UTR
7	Ciclev10019784	24949349	24949949	Mlo family	Intron
8	Ciclev10023902	24986520	24987120	Disease resistance	Intron
9	Ciclev10024232	24991686	24992286	Resistant protein putative	Intron
10	Ciclev10020313	25043084	25043684	Mlo family	Intron
11	Ciclev10022861	25074401	25075001	No annotation	3′UTR
12		25163141	25163741	No gene	–
13		25264697	25265297	No gene	–
14	Ciclev10019183	25443491	25444091	Disease resistance	Exon
15	Ciclev10023260	25495794	25496394	Disease resistance	Exon
16		25861785	25862385	No gen	–
17		26230224	26230824	No gen	–
18		26745996	26746596	No gen	–
19	Ciclev10018492	27236500	27237100	Disease resistance	Exon
20	Ciclev10018528	27868399	27868999	Disease resistance	3′UTR

#### SNP analyses

Polymorphisms found as related to response to the disease were used to develop SNP markers. SNP genotyping was performed by Kbioscience® services, using the KASPar technique. Detailed explanation of specific conditions and reagents can be found in Cuppen (2007).

### Linkage mapping

The mapping population was genotyped with two previously developed SSRs flanking the ABSr locus (Cuenca et al., 2013) and nine new SNP markers developed from the sequences shown in Table 1. Besides, it was phenotyped as described above.

The fine linkage map was constructed using the JoinMap 4.1 software package (Stam, 1993), where markers were coded as *lm* × *ll* (i.e., pseudo-test cross), using Kosambi's mapping function. The recombination threshold was of 0.35, and default values were set for all other parameters. Markers were subjected to segregation distortion analysis.

### Set of parental genotypes to test the new SNP marker selected for MAS

Forty citrus genotypes from the IVIA citrus germplasm bank, either already or candidate to be used as parents in mandarin breeding programs were selected to test the association between the phenotype (resistance to ABS) and the genotype for the closest SNP to the ABS resistance gene (Supplementary Material—Table S1). The set includes 7 citrus species, according to Tanaka's classification (Tanaka, 1977) represented by 12 genotypes and 28 recent hybrids. The selection was done with the aim of spanning the majority of the diversity found within mandarins (Garcia-Lor et al., 2015).

## Results

### SNP mining from sequencing

**Figure 2:**
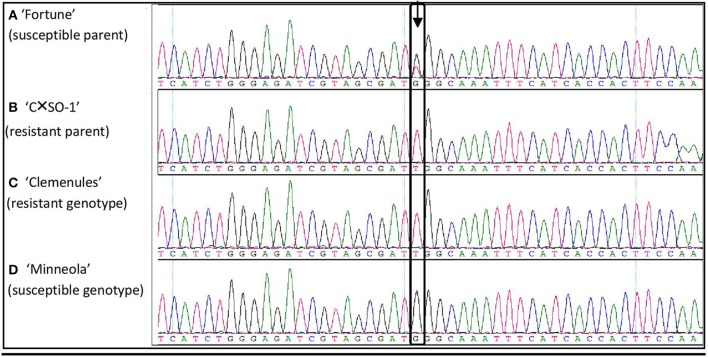
**Sequence alignment of four genotypes revealing the SNP08. (A)** “Fortune”; **(B)** “CxS0-1”; **(C)** “Clemenules”; **(D)** “Minneola.”

With the objective to perform fine mapping, useful SNPs were searched in the previously defined 3.3 Mb region spanning the *ABSr* locus. In a first step, 20 DNA fragments within the *ABSr* region were Sanger-sequenced in the parents of the analyzed population and two additional genotypes (one resistant and one homozygous susceptible) to find informative polymorphisms potentially correlated with the response to the disease. A total of 9.7 kb corresponding to these sequences were finally obtained. Consensus sequences have been submitted to Genbank (accession numbers from KX368965 to KX368984). Twenty-two SNP were found to have useful polymorphisms (data not shown). An example for SNP08 development from sequencing (fragment #16) is shown in Figure 2. At this position, “Fortune” (heterozygous susceptible parent; Figure 2A) is heterozygous G/T and “C × SO-1” (resistant parent; Figure 2B) is homozygous T/T; additional genotypes are “Clemenules” (resistant, T/T; Figure 2C) and “Minneola” (homozygous susceptible G/G; Figure 2D). Finally, nine SNPs included in nine sequenced fragments spanning the entire region were selected to develop KASPar markers for subsequent analysis (Table 2). The absence of additional polymorphisms close to the selected SNP was a major criteria for this second step of selection.

**Table 2:** **Information on new SNP markers developed for fine-mapping and their flanking sequences**.

**SNP**	**Sequence containing the SNP**	**Position in chromosome 3 (Mb)**	**Flanking sequence**
SNP01	#2	24629496	CTGAGTGGATCAGTACAACATAATACTTCTAACAG[A/T]TGCAAAATTTATCACATTAACATTATTAGATTGAT
SNP02	#5	24898391	CCTGGTTCGACAATGCCTCTCTCATTAGTACAACC[A/G]CAATGACAACATATTCGGTCGGCTTGTATGATTCT
SNP03	#7	24949649	GCTTTTAGTGCGCACCATGGCACCGGCCGTCGCCT[T/C]CTAGCTGATGCAGCCTCTGAGGGCAATTGTCCAAA
SNP04	#9	24991986	GCTGTAAAGTGTATCATACACTTTATTAAATTAGG[G/C]TCAATTGGAGTACTGCCGCACACCAATCCTTTGCA
SNP05	#11	25074701	AGACAATCTGTGCATCTTCAAGGCTGAGGAATCTA[A/G]CGGGACAGGCTGATGATTTAGAAAATAACAAACAA
SNP06	#14	25443791	CAAGATTTGATTAAAGTAAAGAAGAAAAAGAAAAA[A/G]GGTAAGAAAAAGTTAGAGAAGATTGAGAAGAAAAA
SNP07	#15	25496094	ACCAAACACTGCAAATTCTTACAGCAGTTTATCTC[G/A]AGGCTTTTAATACATGACGGTAGCTTCTGCCTCGC
SNP08	#16	25862085	ATTTGGCAACCTATCATCTGGGAGATCGTAGCGAT[G/T]GGCAAATTTCATCACCACTTCCAAGTGAAATTTTA
SNP09	#19	27236800	CCTGAAGGGGCATCATCAACTGCTGCTGCTGCACA[C/T]CAGAGGCCACCCAGTTCAAGCGTCCCACCAGAACG

### Fine mapping of the *ABSr* locus

**Figure 3:**
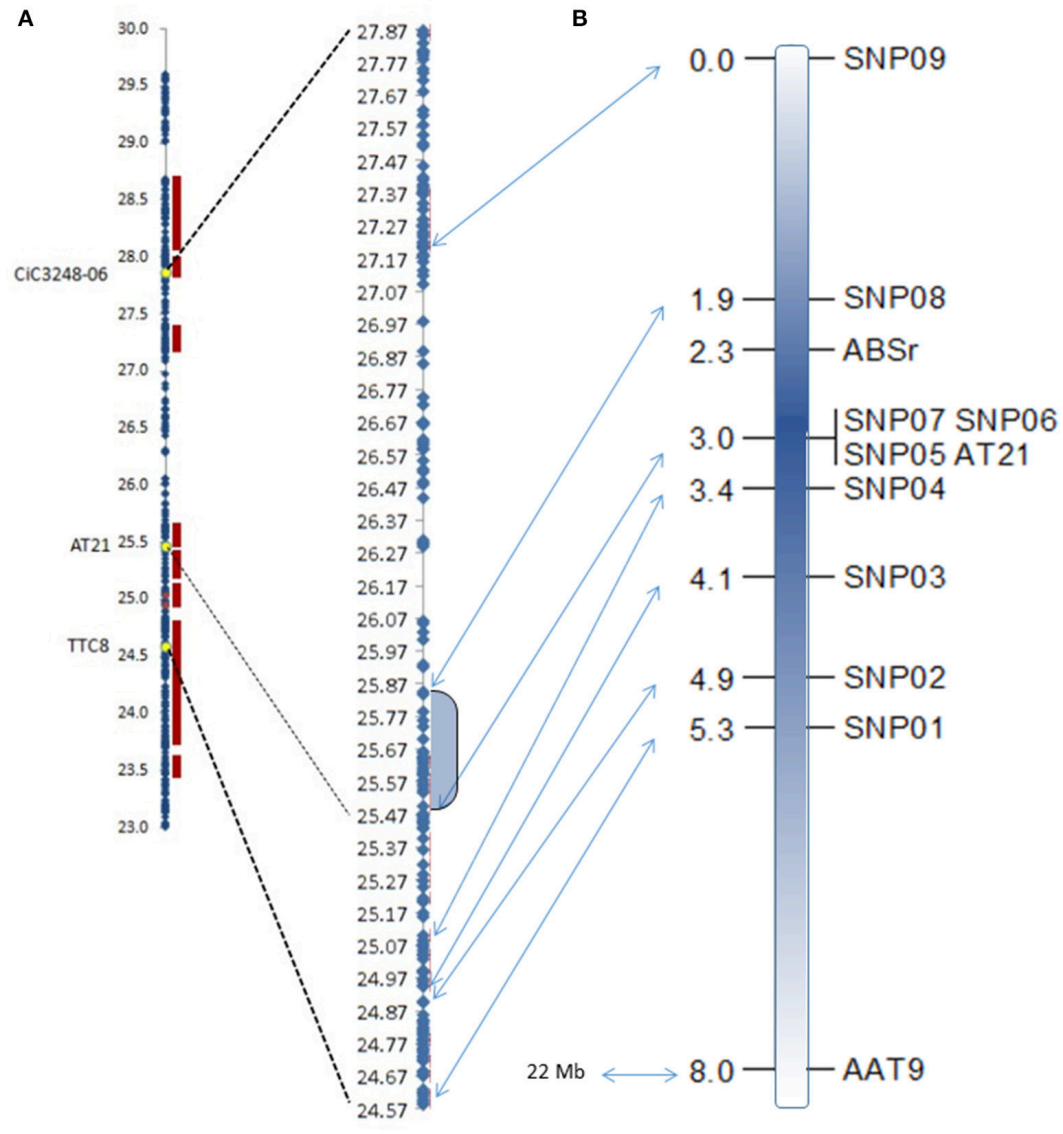
**Physical maps of the ***ABSr*** locus from Cuenca et al. (2013) (A)** compared with the fine genetic mapping of the region from the present study **(B)**. Shadowed area in the physical map corresponds to the identified region flanked by the closest markers. Arrows indicate the physical position of each marker used in the fine mapping. Units are Mbases and centiMorgan in the physical and the genetic maps, respectively.

The new map was constructed using two existing SSR markers and the nine newly developed SNPs markers. Missing data average was 2.0%, ranging from zero for SNP01, SNP02, SNP04, SNP05, SNP06, SNP07, and AT21 markers; 0.37% for SNP08; 0.75% for AAT9, and SNP03 markers, to 20.15% of missing data for the SNP09, (54 individuals). Resistant/susceptible phenotype segregation was 118 (45%)/150 (55%), showing a slightly distorted segregation toward susceptible individuals (χ^2^ = 3.821; *p* = 0.051; n.s.) according to the 1:1 hypothesis. Most of the analyzed markers resulted in similar non-significant bias toward the susceptible genotype. This is a logical observation because all these markers are closely linked. Only the AAT9 (χ^2^ = 5.429; *p* = 0.0198) and the SNP01 (χ^2^ = 4.313; *p* = 0.0378) showed significant distortion at a probability of 95%. Genotyping results for SNP05, SNP06, SNP07, and AT21 markers are identical for all analyzed individuals, so they were localized at the same position in the map. All markers maintained expected mapping order based on their physical location (www.phytozome.org/clementine; Wu et al., 2014). Figure 3 shows the region where the *ABSr* locus is located in the new map compared to the data from Cuenca et al. (2013).

Fine mapping showed that the *ABSr* locus is located in a region of 1.1 cM between the markers SNP05/SNP06/SNP07/AT21 (at 0.7 cM) and SNP08 (at 0.4 cM), limiting the chromosome region to 365.991 bp between the positions 25.496.094 and 25.862.085 within the chromosome 3 in the clementine reference genome sequence. Therefore, this analysis allowed us to narrow the *ABSr* locus to a region containing a much more limited number of candidate genes (Table 3).

**Table 3:** **Annotated genes in the region of interest, indicating their physical position and annotation**.

**Initial position**	**Final position**	**Transcript name**	**Annotation**
25495290	25499648	Ciclev10023260	LRR and NBS-ARC domains-containing disease resistance protein
25539228	25543442	Ciclev10018540	LRR and NBS-ARC domains-containing disease resistance protein
25546089	25546373	Ciclev10023953	Mitochondrial ribosomal protein L11
25558189	25563873	Ciclev10018510	LRR and NBS-ARC domains-containing disease resistance protein
25563004	25563956	Ciclev10024474	No annotation
25577398	25580479	Ciclev10023481	NBS-ARC domain-containing disease resistance protein
25591667	25592171	Ciclev10022922	No annotation
25596184	25598211	Ciclev10019166	No annotation
25598722	25601734	Ciclev10020079	F-box family protein
25605171	25606024	Ciclev10023014	F-box/RNI-like superfamily protein
25612741	25615635	Ciclev10023374	Uridine-ribohydrolase 2
25625682	25630835	Ciclev10019447	Inositol 1,3,4-trisphosphate 5/6-kinase 4
25633452	25636420	Ciclev10018897	Disease resistance protein (CC-NBS-LRR class) family
25639011	25644826	Ciclev10019649	RNA-binding protein
25645230	25649184	Ciclev10021021	Chloroplast outer envelope protein 37
25649848	25653317	Ciclev10024361	S-adenosyl-L-methionine-dependent methyltransferases superfamily protein
25657664	25663490	Ciclev10024293	Endonuclease/exonuclease/phosphatase family protein
25663967	25668616	Ciclev10019293	Ankyrin repeat family protein
25702085	25702716	Ciclev10023674	No annotation
25740008	25740471	Ciclev10023198	Pectin lyase-like superfamily protein
25756520	25759773	Ciclev10018637	Leucine-rich repeat receptor-like protein kinase family protein
25784246	25784449	Ciclev10023998	Mitochondrial pyruvate carrier 2
25838882	25842061	Ciclev10023511	Leucine-rich repeat receptor-like protein kinase family protein
25844828	25845232	Ciclev10024127	Plant self-incompatibility protein S1 family

### Identification of candidate genes for ABS resistance

**Figure 4:**
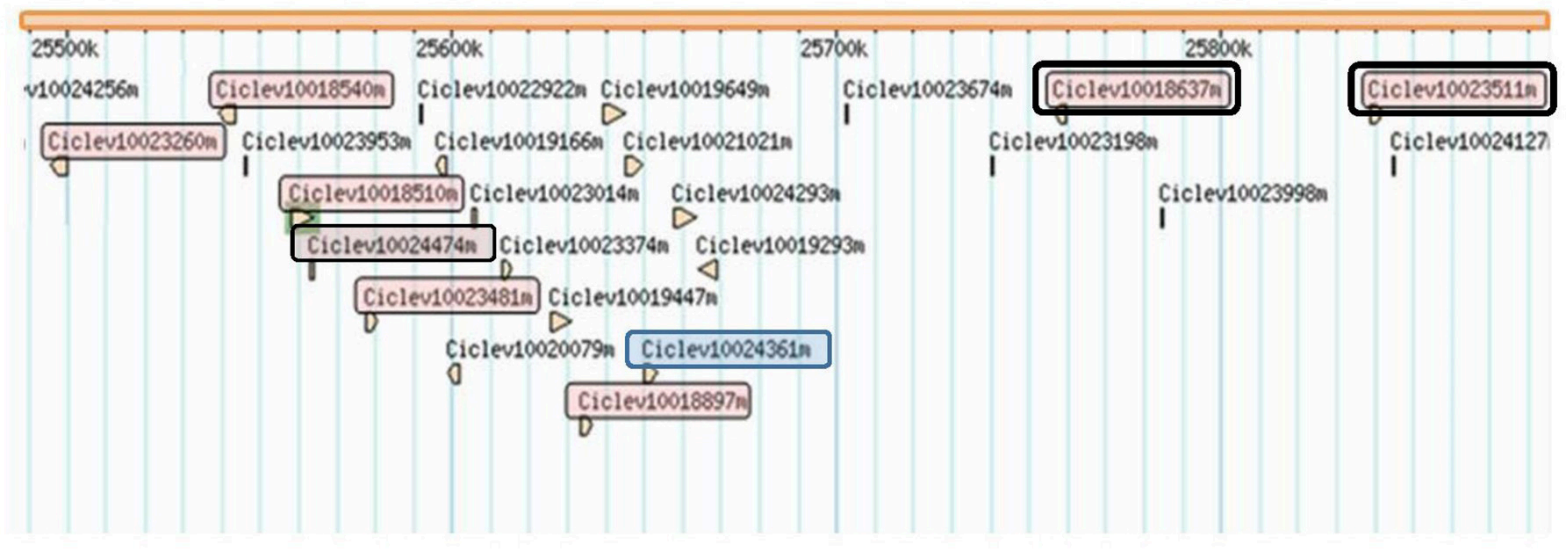
**Physical position of candidate genes for ABS resistance found in the identified mapped within the chromosome 3, between 25.49 and 25.86 Kb**. Genes in red box are NBS-LRR genes; Genes remarked in double box present LRR receptor-like and serine/threonine protein kinase domains; Gene in blue box belongs to the S-adenosyl-L-methionine-dependent methyltransferase family.

Twenty-four genes are predicted in the clementine genome (www.phytozome.org/clementine) within the region delimited by fine-mapping (Table 3; Figure 4). Twenty of these genes have a functional annotation, and eight of them may be related to pathogen resistance. They include seven resistance gene homologs, harboring Leucine-Rich-Repeat (LRR) and Nucleotide-Binding-Site (NBS) domains (Ciclev10023260, Ciclev10018540, Ciclev10018510, Ciclev10023481, Ciclev10018897, Ciclev10018637, and Ciclev10023511). Among these resistance genes, Ciclev10018637 and Ciclev10023511 present LRR receptor-like and serine/threonine protein kinase domains. Another gen (Ciclev10024361) related to pathogen resistance encodes a S-adenosyl-L-methionine-dependent methyltransferases superfamily protein.

In addition to these genes, there are other four genes with no functional annotation in www.phytozome.org website. These genes were then blasted against the NCBI database (https://blast.ncbi.nlm.nih.gov), where no similarity with any previously characterized protein was found for Ciclev10019166, Ciclev10022922, and Ciclev10023674 genes. Blast results for the Ciclev10024474 gene showed high similarity with a predicted putative RPP13-like protein annotated in *Citrus sinensis*. Therefore, this gene was also considered as annotated for disease resistance.

### Response to ABS inoculations in mandarin breeding parents

Detached leaves from 40 genotypes used or selected to be used as parents in mandarin breeding programs were inoculated with *A. alternata* spores as explained in Materials and Methods Section. Results indicate that 20 of them showed symptoms after inoculations, scored as susceptible varieties; 20 varieties did not show any symptom in any replicate, and then were scored as resistant genotypes. Results of ABS response are in agreement with most of the previous studies for all the genotypes for which information is available (Table 4).

**Table 4:** **ABS response, SNP08 genotyping results, and previous data for ABS response for the 40 cultivars analyzed in the present study**.

**Group**	**Cultivar**	**ABS response**	**SNP08 genotyping**	**Previous results for ABS response**
Clementine	Clemenules	Resistant	TT	*Resistant*: Hutton and Mayers, 1988; Kohmoto et al., 1991; Solel and Kimchi, 1997; Vicent et al., 2004; Dalkilic et al., 2005; Elena, 2006; de Souza et al., 2009; Kakvan et al., 2012; Pacheco et al., 2012; Cuenca et al., 2013
Mandarin	Anana	Resistant	TT	
Mandarin	Campeona	Resistant	TT	*Resistant*: Vicent et al., 2004
Mandarin	Carvahal	Resistant	TT	
Mandarin	Dancy	Susceptible	GG	*Susceptible:* Whiteside, 1979; Solel and Kimchi, 1997
Mandarin	Emperor	Susceptible	GT	*Susceptible:* Pegg, 1966; Vicent et al., 2004
Mandarin	King	Resistant	TT	*Resistant:* de Souza et al., 2009; **Susceptible:** Solel and Kimchi, 1997
Mandarin	Ponkan	Susceptible	GT	*Susceptible:* Pacheco et al., 2012
Mandarin	Scarlet	Resistant	TT	
Mandarin	Temple	Resistant	TT	*Resistant*: Hutton and Mayers, 1988
Mandarin	Willowleaf	Resistant	TT	*Resistant*: Hutton and Mayers, 1988; Solel and Kimchi, 1997; Vicent et al., 2004; Cuenca et al., 2013
Mandarin hybrid	Daisy	Susceptible	GG	*Susceptible:* de Souza et al., 2009; Stuart et al., 2009
Mandarin hybrid	Encore	Resistant	TT	*Resistant:* Kohmoto et al., 1991; de Souza et al., 2009
Mandarin hybrid	Fairchild	Susceptible	GT	*Susceptible:* Vicent et al., 2004; Dalkilic et al., 2005; Cuenca et al., 2013
Mandarin hybrid	Fallglo	Susceptible	GT	
Mandarin hybrid	Fortune	Susceptible	GT	*Susceptible:* Vicent et al., 2004; Dalkilic et al., 2005; Kakvan et al., 2012; Cuenca et al., 2013
Mandarin hybrid	Fremont	Susceptible	GG	*Susceptible:* Pacheco et al., 2012; **Resistant:** de Souza et al., 2009
Mandarin hybrid	Gold Nugget	Resistant	TT	
Mandarin hybrid	Honey	Resistant	TT	
Mandarin hybrid	Kara	Resistant	TT	*Resistant:* Hutton and Mayers, 1988; Kohmoto et al., 1991; Vicent et al., 2004; **Susceptible:** Solel and Kimchi, 1997
Mandarin hybrid	Kinnow	Resistant	TT	
Mandarin hybrid	Moncada	Resistant	TT	
Mandarin hybrid	Nova	Susceptible	GT	*Susceptible:* Solel and Kimchi, 1997; Vicent et al., 2004; Dalkilic et al., 2005; Elena, 2006; Pacheco et al., 2012; Cuenca et al., 2013
Mandarin hybrid	Osceola	Susceptible	GT	
Mandarin hybrid	Page	Susceptible	GT	*Susceptible:* Solel and Kimchi, 1997; Vicent et al., 2004; Dalkilic et al., 2005; Elena, 2006; Kakvan et al., 2012; Pacheco et al., 2012
Mandarin hybrid	Palazzelli	Resistant	TT	
Mandarin hybrid	Pixie	Susceptible	GT	
Mandarin hybrid	Primosole	Susceptible	GT	
Mandarin hybrid	Simeto	Resistant	TT	*Resistant:* Vicent et al., 2004
Mandarin hybrid	Sunburst	Susceptible	GT	*Susceptible:* Pacheco et al., 2012
Mandarin hybrid	Wilking	Resistant	TT	
Satsuma	Frost	Resistant	TT	*Resistant*: Vicent et al., 2004; de Souza et al., 2009; **Susceptible:** Solel and Kimchi, 1997
Tangelo	Mapo	Resistant	TT	Vicent et al., 2004
Tangelo	Minneola	Susceptible	GG	*Susceptible:* Whiteside, 1979; Solel and Kimchi, 1997; Peever et al., 1999; Vicent et al., 2004; Dalkilic et al., 2005; Elena, 2006; Kakvan et al., 2012; Cuenca et al., 2013
Tangelo	Orlando	Susceptible	GT	*Susceptible:* Whiteside, 1979; Solel and Kimchi, 1997; Dalkilic et al., 2005; Kakvan et al., 2012; Cuenca et al., 2013
Tangelo	Seminole	Susceptible	GT	
Tangor	CxSO-1	Resistant	TT	
Tangor	Dweet	Susceptible	GT	
Tangor	Ellendale	Susceptible	GT	*Susceptible:* Solel and Kimchi, 1997; *Resistant***:** Vicent et al., 2004
Tangor	Murcott	Susceptible	GT	*Susceptible:* Solel and Kimchi, 1997; Vicent et al., 2004; Dalkilic et al., 2005; de Souza et al., 2009; Pacheco et al., 2012; Cuenca et al., 2013

### New reliable SNP marker for MAS in mandarin breeding programs

The closet SNP marker flanking the ABSr locus was SNP08, mapped at 0.4 cM from the responsible gene for ABS resistance. This marker was analyzed using the KASPar technique in 40 citrus genotypes, including 20 resistant, and 20 susceptible cultivars. Results from phenotyping and SNP08 genotyping for these 40 accessions are indicated in Table 4. As previously identified from the segregating populations, the allele of this SNP marker linked with the dominant susceptible allele of the ABSr locus is *G*. Therefore, considering identity by descent in the analyzed germplasm, without recombination between the marker and the ABSr locus, resistant genotypes are expected to be *TT*, whereas susceptible genotypes are expected to be *GG* or *GT*.

**Figure 5:**
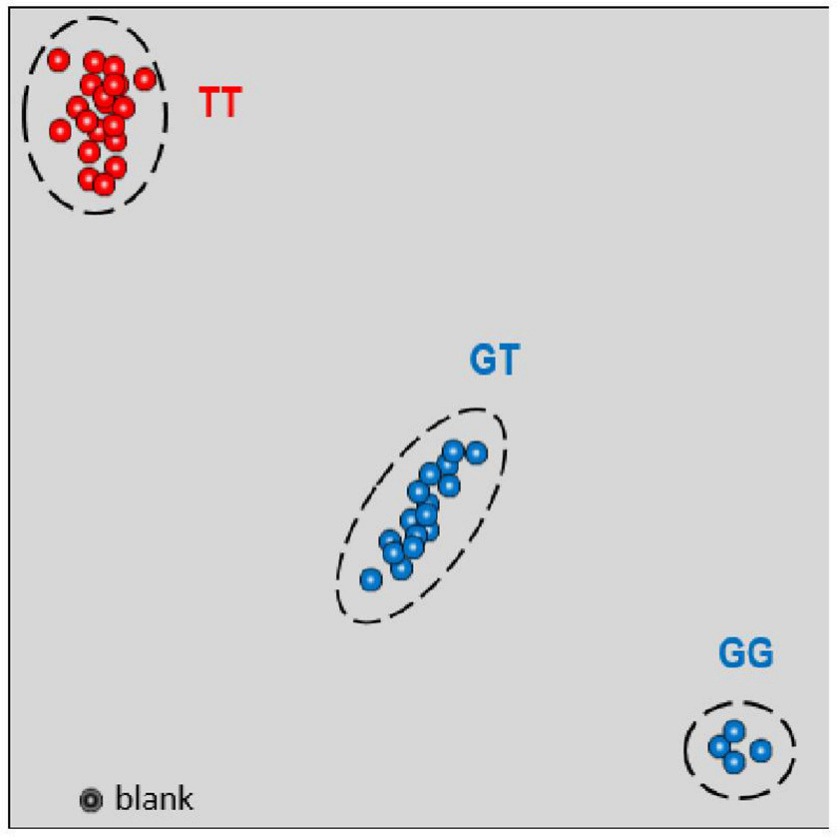
**KASPar genotyping results for the SNP08 for the set of genotypes selected for this study**. Resistant cultivars are represented by red points (TT); susceptible cultivars are represented by blue points (GT and GG).

All genotypes included in the study were correctly classified as resistant or susceptible using this single SNP marker, highlighting its discriminating power between the two groups (Figure 5).

## Discussion

ABS is an important fungal disease affecting susceptible citrus genotypes. Currently, ABS control is primarily based on fungicide application, but despite a large number of sprays, disease control is not always satisfactory and cannot be considered as a sustainable crop management practice. As a consequence, cultivation of susceptible cultivars has declined significantly, as is the case of “Fortune” mandarin in Spain during recent years. Thus, genetic resistance remains as the best option for disease control [1]. The objective of this study was (i) to finely map the *ABSr* locus identified by Cuenca et al. (2013) on LG III of the clementine's genetic map (Ollitrault et al., 2012), (ii) to identify candidate genes for resistance, and (iii) to develop SNP molecular markers for efficient MAS in citrus breeding programs.

### A 366 Kb genomic region has been targeted for alternaria brown spot resistance in citrus

In our previous work (Cuenca et al., 2013), the *ABSr* locus was located by using a bulked segregant analysis coupled with genome scan and targeted genetic mapping by half tetrad analysis in a triploid progeny. The region containing the ABSr locus was flanked by markers TTC8 and CiC3248-06 defining a 5.48 cM interval, corresponding to 3.3 Mb in the clementine reference genome. In the present work, the fine-mapping was carried out using a 268-diploid progeny arising from a heterozygous susceptible × resistant hybridization. As a result, we have limited the candidate region containing the *ABSr* locus to 1.5 cM flanked by two SNP markers at 1.1 and 0.4 cM, corresponding to 366 kb in the clementine reference genome (between positions 25.496.094 and 25.862.085 in the chromosome III).

### Candidate genes for ABS resistance

Interactions between pathogens and their hosts are complex and dynamic. At the early stages of necrotrophic infections, host cell death is associated with the production of various secondary metabolites, antimicrobial peptides, hormones and also with the accumulation of reactive oxygen species, callose, and some other cell wall modifications (Wen, 2012). Cell death is a common phenomenon in both resistant and susceptible responses of plant–pathogen interactions to confine pathogens by abolishing nutrient supply. However, cell death generally enhances colonization by necrotrophic pathogens. Effectors produced by the fungus can be recognized by immune receptors in plants, generally belonging to the nucleotide–binding site–leucine–rich repeat (NBS–LRR) protein family. The identified region contains eight genes harboring NBS-LRR repeats, which should be considered as candidate genes for ABS resistance. Nevertheless, the *RLM3*, a Toll/interleukin 1 receptor domain R–protein in *Arabidopsis* is the only NBS-LRR gene found to be responsible for resistance to necrotrophs (Mengiste, 2012). A BLAST with this protein results in 200 hits in the citrus genome. Surprisingly, 170 of them are located within chromosome 3, showing the highest score (E = 8.1e-58), and 37% of them are physically mapped around the region of interest (i.e., from 21 to 28 Mb in chromosome 3); however, none of them is within the region identified by fine mapping.

Among the identified resistance genes, Ciclev10018637 and Ciclev10023511 encode for a Leucine-Rich Repeat (LRR) receptor-like protein with serine/threonine kinase domain. LRR receptor-like kinases (LRR-RLK) appear to play a central role in signaling during pathogen recognition, the subsequent activation of plant defense mechanisms, and developmental control (Afzal et al., 2008). Indeed, most of the RLK identified as being involved in plant defense are of the LRR-RLK class including the rice Xa21 protein and the Arabidopsis FLS2 and EFR receptors (Goff and Ramonell, 2007). Both genes were mapped very close to the most significant SNP related to ABS resistance (SNP08). Thus, they should be considered as strong candidates for resistance.

Another strong candidate for ABS resistance found within the region of interest and close to the SNP08 is the gene Ciclev10024361, encoding for an S-adenosyl-L-methionine-dependent methyltransferases superfamily protein, with thiopurine S-methyltransferase superfamily protein. The ACTTS3 gene from *A. alternata*, involved in the production of the ACT-toxin, encodes a polyketide synthase with putative β-ketoacyl synthase, acyltransferase, methyltransferase, β-ketoacyl reductase, and phosphopantetheine attachment site domains (Miyamoto et al., 2010). During infections of *Brassica juncea* with *Alternaria brassicola*, the transcriptional activation of jasmonic acid carboxyl methyltransferase was observed after 2 days post infection (Meur et al., 2015). This gene could be a good target for achieving resistance against necrotrophic pathogens, and therefore, it should also be considered as a strong candidate for resistance to ABS.

There is little information in other plant species about genes involved in resistance to necrotrophic pathogens in general, and to *A. alternata* in particular. In *Solanum lycopersicum*, the *Asc* gene mediates resistance to AAL toxin produced by *A. alternata* lycopersici (Spassieva et al., 2002). A BLAST for this gene in citrus indicates that there are orthologous genes in two regions of chromosome 3, but none of them within the region of interest; in addition, many other regions in other chromosomes contain *Asc* orthologous genes. In barley, *mlo* mutants are resistant to penetration by *Blumeria graminis*, overproducing p-coumaroyl-hydroxyagmatine; this compound has antifungal activity and inhibits haustorium formation *in vivo* (Büschges et al., 1997; von Röpenack et al., 1998). BLAST for *mlo* family genes in citrus indicates that seven of the nine citrus chromosomes contain orthologous to this family, including three genes found to be very close (but outside) to the region of interest in chromosome 3 (Ciclev10019784, Ciclev10023336, Ciclev10020313). In *Arabidopsis*, Coego et al. (2005) showed that the homeodomain transcription factor *OCP3*, mediates resistance to infection by necrotrophic pathogens. BLAST results for this gene showed that the only orthologous gene in the citrus genome (Ciclev10010768) is in the chromosome 1. Arabidopsis *RFO1* gene, conferring quantitative resistance against *Fusarium oxysporum* (Diener and Ausubel, 2005), had significant BLAST in five regions of chromosome 3, but none of them within the region of interest; in addition, many other regions in other chromosomes contain *RFO1* orthologous genes. Other genes and transcriptions factors have been found to be implicated in the resistance to necrotrophs in *Arabidopsis*, like the *BOS1* and *BOI*, which appear to restrict necrosis triggered by diverse pathogens and stress factors (Luo et al., 2010). BLAST for these genes in citrus reveals orthologous genes in chromosomes 2 and 3, but outside the region of interest. The WRKY33 transcription factor is required for resistance to necrotrophic fungal pathogens in Arabidopsis (Zheng et al., 2006) and BLAST results in citrus indicate that there are orthologous genes in chromosome 3, but outside the region of interest. Also, the *ATG18a* gene, which interacts with WRKY33, has no orthologous within the region of interest.

The information generated from sequence comparison and annotations allows to discard 15 out of the 24 genes found within the region delimited by fine-mapping. Among the nine genes that may be related to pathogen resistance, the genes Ciclev10018637, Ciclev10023511, and Ciclev10024361 are the strongest candidates for ABS resistance in citrus. This limited number will now allow to make affordable although time-consuming approaches to determine whether these genes are really involved in the *Alternaria*-citrus interaction. Further experiments, including differential expression performed during fungal infection and functional approaches as genetic transformation or reverse genetics should be performed to accomplish this goal.

### A new SNP marker for efficient MAS has been developed

In fruit crops, several molecular markers have been found to be linked to agronomic traits of interest (van Nocker and Gardiner, 2014). Examples of successful early selection of interesting genotypes within breeding programs are found in grapes (www.vitisgen.org), *Prunus* and other *Rosaceae* species (Dirlewanger et al., 2004).

In citrus, a few characters of agronomic interest have been linked to molecular markers, such as RAPD markers linked to dwarfing (Cheng and Roose, 1995) or fruit acidity (Fang et al., 1997), SSR markers linked to CTV resistance from *Poncirus trifoliata* (Gmitter et al., 1996; Bernet et al., 2004), AFLP markers linked to nucellar embryony (García et al., 1999; Kepiro and Roose, 2010), and the dominant PCR assay for the anthocyan content of pulp from blood orange due to a transposable element in the 5′ extremity of the Ruby gene (Butelli et al., 2012). Other characters of interest have been tagged to QTLs, such as salinity tolerance (Ben-Hayyim and Moore, 2000) and nematode resistance (Ling et al., 2000). However, for citrus scion breeding programs, only the *Alternaria* resistance presented in this paper, along with the anthocyanin content, are currently subjected to MAS.

ABS resistance has also been previously tagged with molecular markers. Dalkilic et al. (2005) reported two RAPD markers with loose linkage with the locus (15.3 and 36.7 cM far from ABS resistance locus in the same side). More recently, Gulsen et al. (2010) identified two flanking SRAP markers at 3 and 13 cM. However, due to the distance between markers and the resistance gene as well as the dominant characters of RAPDs and SRAP, these studies did not resulted in concrete MAS application.

In the present work, we have developed a SNP marker (SNP08) mapped at 0.4 cM from the ABS resistance gene, which greatly improves the selection of resistant genotypes in early development stages and avoid growing and evaluating susceptible genotypes. The SNP08 marker is diallelic (G/T), in which the G base is phased with ABS dominant susceptibility. It means that when the G allele is present, i.e., GT and GG genotypes, the cultivar is expected to be susceptible and therefore, only TT genotypes are expected to be resistant. With 0.4 cM distance, in a progeny between a heterozygous susceptible parent and a resistant one, only 0.4% of GT hybrids are expected to be resistant and the same proportion is expected for TT hybrids to be susceptible. Flanking markers at 0.7 cM from the *ABSr* locus have also been identified on the other side of the gene. By coupling the SNP08 and one of these markers, the error rate would fall to 0.0028% (<3 false resistant hybrids per 100.000).

This SNP08 marker has been tested for association in 40 mandarin genotypes used as breeding parents and covering a large range of the mandarin diversity (Garcia-Lor et al., 2015). ABS inoculations revealed 20 resistant and 20 susceptible genotypes. *Citrus clementina, C. nobilis, C. temple, C. deliciosa*, and *C. unshiu* species resulted resistant, whereas the analyzed genotype from *C. tangerina* is susceptible. *Citrus reticulata* and the group of hybrid mandarins include both resistant and susceptible genotypes. In the latter group, hybrids between resistant genotypes are resistant, in agreement with the recessive inheritance of the ABS resistance.

Since SNP08 is very close to the ABS resistance gene, it is expected to obtain a very close correlation between the observed SNP08 genotyping and the actual allele configuration of the resistance gene in the germplasm. However, the development of this new marker is based on the segregation of only one progeny, and, *a priori*, the transferability between different citrus species or interspecific hybrids may not be efficient. In this work, we confirmed this transferability by comparing the results observed for some varieties with segregation progenies obtained from previous works. GG genotypes for the SNP08 are susceptible presumably homozygous for the gene, and no segregation is expected in their progeny. This has been demonstrated for “Minneola” by Cuenca et al. (2013), from which no resistant genotypes were obtained in a progeny of 127 hybrids. Moreover, segregations in the response to ABS were observed by the same authors in progenies arising from “Fortune,” “Nova,” “Orlando,” “Fairchild,” and “Murcott,” confirming that all these cultivars (genotyped as “GT” for the SNP08) are heterozygous for the *ABSr* locus. Therefore, this marker appears to be phased with the ABS resistance gene in the analyzed germplasm and it could be very useful not only for discriminating between resistant and susceptible cultivars, but also for inferring the allele configuration of the ABS resistance gene. It is therefore a valuable tool for selecting susceptible heterozygous genotypes that can be used as parents in breeding programs and conversely to discard homozygous susceptible varieties. The fact that susceptible yet heterozygous genotypes can be used as parents allows the exploitation of more genetic diversity in citrus breeding programs.

The SNP08 is currently used for the assisted selection of ABS-resistant genotypes in the citrus breeding programs carried out at IVIA and CIRAD. Since its development, it has been successfully applied to select 2187 resistant hybrids from 4517 total hybrids arising from 10 different parental combinations. This analysis avoided to keep growing for a long time more than 2000 susceptible genotypes, which were removed from the breeding program at a very early stage of development and, therefore, saving a considerable amount of time, personnel and money resources.

## Author contributions

JC performed the experiments, analyzed the data, and wrote the manuscript. AG performed molecular experiments. PA obtained and provided the plant material. PO and LN conceived and supervised the experiments. All authors revised and approved the manuscript.

## Funding

This work was supported by a grant [AGL2011-26490] from the Ministry of “Economía y Competividad”—Fondo Europeo de Desarrollo Regional (FEDER) and a grant [Prometeo 2008/121] from the Generalitat Valenciana, Spain.

### Conflict of interest statement

The authors declare that the research was conducted in the absence of any commercial or financial relationships that could be construed as a potential conflict of interest.
