# Supplementary material for: Fine Mapping for Identification of Citrus Alternaria Brown Spot Candidate Resistance Genes and Development of New SNP Markers for Marker-Assisted Selection
Source: Front Plant Sci. 2016 Dec 23;7:1948. doi: 10.3389/fpls.2016.01948 (PMC5179576; doi:10.3389/fpls.2016.01948)
Supplement: Supplementary file 1 [file Table1.DOCX]

Supplementary Material

Fine mapping for identification of Citrus Alternaria Brown Spot candidate resistance genes and development of new SNP markers for marker-assisted selection

Jose Cuenca^1^, Pablo Aleza^1^, Andres Garcia-Lor^1^, Patrick Ollitrault^2*^, Luis Navarro^1*^

*** Correspondence:** Luis Navarro [lnavarro@ivia.es](mailto:lnavarro@ivia.esP) Patrick Ollitrault [patrick.ollitrault@cirad.fr](mailto:patrick.ollitrault@cirad.fr)

# Supplementary Tables

Table S1. Set of cultivars used to test the new marker selected for MAS, indicating the accession number from IVIA germplasm, latin name and commercial group.

| Group | Genotypes | IVIA Accession number | Latin name (Tanaka classification) |
| --- | --- | --- | --- |
| Clementine | Clemenules | 22 | *C. clementina* |
| Mandarin | Anana | 390 | *C. reticulata* |
| Mandarin | Campeona | 193 | *C. nobilis* |
| Mandarin | Carvahal | 568 | *C. reticulata* |
| Mandarin | Dancy | 434 | *C. tangerina* |
| Mandarin | Emperor | 394 | *C. reticulata* |
| Mandarin | King | 477 | *C. nobilis* |
| Mandarin | Ponkan | 482 | *C. reticulata* |
| Mandarin | Scarlet | 411 | *C. reticulata* |
| Mandarin | Temple | 81 | *C. temple* |
| Mandarin | Willowleaf | 154 | *C. deliciosa* |
| Mandarin hybrid | Daisy | 362 | *(C. clementina x C. tangerina) x (C. clementina x C. reticulata)* |
| Mandarin hybrid | Encore | 155 | *C. nobilis x C. deliciosa* |
| Mandarin hybrid | Fairchild | 83 | *C. clementina x (C. paradisi x C. tangerina)* |
| Mandarin hybrid | Fallglo | 466 | [*C.clementina*x (*C. paradisi*x *C. tangerina)*] x *C. temple* |
| Mandarin hybrid | Fortune | 80 | *C. clementina x C. tangerina* |
| Mandarin hybrid | Fremont | 82 | *C. clementina x C. reticulata* |
| Mandarin hybrid | Gold Nugget | 523 | *(C. deliciosa x ?) x (? x C. tangerina)* |
| Mandarin hybrid | Honey | 209 | *C. nobilis x C. deliciosa* |
| Mandarin hybrid | Kara | 218 | *C. unshiu x C. nobilis* |
| Mandarin hybrid | Kinnow | 33 | *C. nobilis x C. deliciosa* |
| Mandarin hybrid | Moncada | 421 | *C clementina x (C. unshiu x C. nobilis)* |
| Mandarin hybrid | Nova | 74 | *C. clementina x (C. paradisi x C. tangerina)* |
| Mandarin hybrid | Osceola | 573 | *C. clementina x (C. paradisi x C. tangerina)* |
| Mandarin hybrid | Page | 79 | *(C. paradisi x C. tangerina) x C. clementina* |
| Mandarin hybrid | Palazzelli | 188 | *C. clementina x C. nobilis* |
| Mandarin hybrid | Pixie | 210 | *(C. nobilis x C. tangerina) x ?* |
| Mandarin hybrid | Primosole | 414 | *C. unshiu x (C. deliciosa x ?)* |
| Mandarin hybrid | Simeto | 413 | *C. unshiu x C. deliciosa* |
| Mandarin hybrid | Sunburst | 200 | *[C. clementina x (C. paradisi x C. tangerina)] x [C. clementina x (C. paradisi x C. tangerina)]* |
| Mandarin hybrid | Wilking | 28 | *C. nobilis x C. deliciosa* |
| Satsuma | Frost | 175 | *C. unshiu* |
| Tangelo | Mapo | 190 | *C. deliciosa x C. paradisi* |
| Tangelo | Minneola | 84 | *C. paradisi x C. tangerina* |
| Tangelo | Orlando | 101 | *C. paradisi x C. tangerina* |
| Tangelo | Seminole | 348 | *C. paradisi x C. tangerina* |
| Tangor | CxSO-1 | - | *C. clementina x C. sinensis* |
| Tangor | Dweet | 165 | *C. tangerina x C. sinensis* |
| Tangor | Ellendale | 194 | *C. reticulata x C. sinensis* |
| Tangor | Murcott | 196 | *C. reticulata x C. sinensis* |
